# The neutrophil-to-lymphocyte ratio is associated with the frequency of delayed neurologic sequelae in patients with carbon monoxide poisoning

**DOI:** 10.1038/s41598-023-47214-5

**Published:** 2023-11-11

**Authors:** Dawei Xu, Tianshu Mei, Fei He

**Affiliations:** 1 Department of Emergency Medicine, the Affiliated Suqian Hospital of Xuzhou Medical University, Suqian, 223800 China; 2 Department of Emergency Medicine, Nanjing Drum Tower Hospital, Affiliated Hospital of Medical School, Nanjing University, Nanjing, 210008 China

**Keywords:** Biomarkers, Health occupations, Medical research, Neurology, Risk factors

## Abstract

Delayed neurologic sequelae (DNS) is a common complication in patients with carbon monoxide poisoning (COP). We aimed to investigate the association of the neutrophil-to-lymphocyte ratio (NLR) with the frequency of DNS in COP patients. A total of 371 COP patients were investigated in retrospective and prospective studies. A receiver operator curve (ROC) test was performed to evaluate the ability of the NLR to predict DNS in COP patients. The retrospective study included 288 COP patients, of whom 84 (29.2%) were confirmed to have DNS, and 1 (0.3%) died within 28 days. The NLR in the DNS group was significantly higher than that in the non-DNS group (6.84 [4.22–12.43] vs. 3.23 [1.91–5.60] × 10^9^/L). NLR was a significant predictor of the frequency of DNS [odds ratio (OR): 1.130, 95% confidence interval (CI): 1.030, 1.240] in COP patients. The area under the ROC curve of NLR for predicting DNS was 0.766 (95% CI 0.701, 0.832), and the cut-off value was 3.745 (sensitivity, 83.3%; specificity, 58.8%). The prospective study included 83 COP patients, of whom 19 (22.9%) were confirmed to have DNS, and all patients survived. Moreover, the frequency of DNS in the patients with an NLR ≥ 3.745 was notably higher than that in the patients with an NLR < 3.745 [41.4% (12/29) vs. 13.0 (7/54)]. In conclusion, the NLR was a significant, independent predictor of the frequency of DNS in COP patients.

## Introduction

Carbon monoxide poisoning (COP) is considered one of the major health concerns that can lead to neurological sequelae or even death in clinical toxicology. The clinical manifestations of this condition include headache, asthenia, fatigue, nausea/vomiting, altered mental status, chest pain, breathlessness, and transient loss of consciousness, with a mortality ranging from 1 to 3%^1^. In addition, long-term neurological and neuropsychiatric sequelae associated with cerebral injury, described as delayed neurologic sequelae (DNS), occur in 15–40% of patients who survive COP^1^. The symptoms of DNS consist of personality changes, depression, memory loss, language impairment, movement disorders, and focal neurologic deficits, occurring from several days to months after COP^2^. It has a profound effect on the quality of life of patients and their families. Therefore, the exploration of possible predictors is important for identifying patients at risk of DNS after COP and for guiding early management strategies to improve neurological prognosis in patients with COP. Currently, various epidemiological, laboratory, and clinical indicators can be used to efficiently predict the neurologic outcomes of COP patients^3–6^. However, some of them are inconvenient to obtain, and it is difficult to evaluate those parameters freely during clinical practice.

The neutrophil-to-lymphocyte ratio (NLR) is a peripheral blood-derived inflammation marker that reflects the balance between systemic inflammation and immunity^7^. It serves as an inexpensive, easily available, and effective predictive marker for adverse outcomes in patients suffering from acute medical conditions, such as sepsis^8^, acute pancreatitis^9^ and infectious disease^10^. Meanwhile, the NLR plays a key role in the prediction of the prognosis of patients with neurological injury disease. A retrospective observational cohort study conducted by Chen CT et al. revealed that the NLR was an independent factor predicting the neurological outcome of patients with acute ischaemic stroke, and a lower NLR was associated with a favourable neurologic outcome in serial mid- and long-term follow-up^11^. A previous study revealed that excess CO generates reactive oxygen species (ROS) and nitric oxide (NO); the inflammation induced by NO and ROS contributes to neurological injuries from CO poisoning^1^. Nevertheless, little is known about the relationship between the NLR and the frequency of DNS in patients with COP.

Therefore, in this study, we aimed first to investigate the relationship between the NLR and the frequency of DNS in patients with COP. Then, we sought to verify the predictive value of a higher NLR level for the frequency of DNS in patients with COP. We speculated that a higher NLR was a novel biomarker in peripheral blood indicating a poor neurologic outcome in patients with COP.

## Results

**Figure 1:**
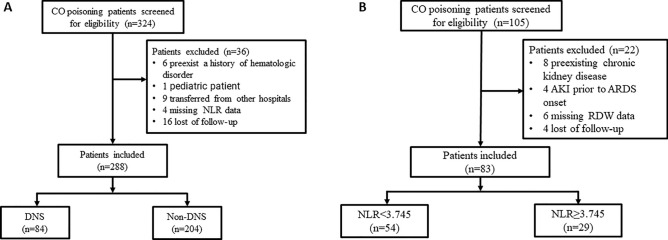
Flow chart of the study participants. (**A**) retrospective cohort; (**B**) prospective cohort.

A total of 324 patients diagnosed with COP were eligible for the retrospective study, of whom 288 patients met the inclusion criteria (Fig. 1A). The median age of the COP patients was 53.48 ± 19.29 years, and 40.3% (116/288) were male. Overall, the frequency of DNS was 29.2% (84/288), and the 28-day mortality was 0.3% (1/288). A comparison of baseline demographic and clinical characteristics and laboratory parameters between patients in the DNS and non-DNS groups is shown in Table 1. In the DNS group, patients showed significantly higher values for age, proportion of males, TWBC count, NLR, ALT, AST, SCr, BUN and serum lactate than those in the non-DNS group. Moreover, the frequency of AKI and ARDS and the proportion of HFNC in the DNS group were notably higher than those in the non-DNS group. However, the GCS score in the DNS group was notably lower than that in the non-DNS group. In addition, there was no significant difference in 28-day mortality or length of hospital stay between the two groups.

**Table 1:** Baseline characteristics and laboratory parameters of the DNS and Non-DNS groups.

Variable	Total (n = 288)	DNS (n = 84)	Non-DNS (n = 204)	*P* Value
Demographics				
Age, mean ± SD, years	53.48 ± 19.29	59.37 ± 20.00	51.05 ± 18.50	0.001
Male, sex, n (%)	116 (40.3)	47 (56.0)	69(33.8)	0.001
Smoking, n (%)	79 (27.4)	26(31.0)	53(26.0)	0.390
Suicide attempt, n (%)	4 (1.4)	1 (1.2)	3 (1.5)	1.000
Unintentional intoxication, n (%)	284(98.6)	83(98.8)	201(98.5)	0.854
Time to ED visit, median (IQR), hours	8 (5.0,10.0)	7.0(4.0,10.0)	8(5.0,11.75)	0.055
Chronic comorbidities, n (%)				
Hypertension	56 (19.4)	21(25.0)	35(17.2)	0.126
Diabetes mellitus	12 (4.2)	6(7.1)	6(2.9)	0.105
CAD	9 (3.1)	5(6.0)	4(2.0)	0.162
COPD	12(4.2)	6(7.1)	6(2.9)	0.105
GCS score, mean ± SD	9.43 ± 3.30	6.57 ± 2.12	10.61 ± 2.96	< 0.001
MAP, mean ± SD, mmHg	96.71 ± 59.20	105.47 ± 105.70	93.10 ± 18.48	0.289
Laboratory parameters				
TWBC count, mean ± SD, × 10^9^/L	9.28 ± 3.97	11.26 ± 4.37	8.46 ± 3.49	< 0.001
NLR, median (IQR), × 10^9^/L	4.21 (2.38,7.27)	6.84 (4.22,12.43)	3.23(1.91,5.60)	< 0.001
HCT, mean ± SD, %	38.70 ± 5.06	38.22 ± 5.74	38.98 ± 4.75	0.349
ALT, median (IQR), U/L	19.45 (14.9,27.0)	22.05 (16.43,34.00)	18.95 (14.0,26.0)	0.005
AST, median (IQR), U/L	22.9 (17.1,29.85)	28.6 (21.65,50.68)	21.0 (16.43,27.0)	< 0.001
SCr, mean ± SD, umol/L	62.50 ± 18.08	74.89 ± 20.02	57.39 ± 14.43	< 0.001
BUN, mean ± SD, mmol/L	5.95 ± 2.10	6.83 ± 2.36	5.58 ± 1.87	< 0.001
Lactate, median (IQR), mmol/L	2.0 (1.5,2.88)	2.35 (1.73,3.60)	1.8 (1.4,2.5)	0.001
COHb, mean ± SD, (%)	29.15 ± 12.53	30.92 ± 13.14	28.35 ± 12.58	0.239
Complications, n (%)				
AKI	86 (29.9)	56 (66.7)	30 (14.7)	< 0.001
ARDS	8(2.8)	8(9.5)	1(0.5)	< 0.001
Interventions, n (%)				
HFNC	18 (6.3)	13(15.5)	5(2.5)	< 0.001
IMV	2(0.7)	0(0)	2(1.0)	0.896
HBOT	225 (78.1)	62 (73.8)	163 (79.9)	0.256
Outcomes				
28-day mortality, n (%)	1 (0.3)	1 (1.2)	0 (0)	0.646
Hospital stays, median (IQR) days	8.00 (5.00,10.00)	7.0 (4.0,10.0)	8.0 (5.0,11.75)	0.055

AST, aspartate aminotransferase; ALT, alanine aminotransferase; AKI, acute kidney injury; ARDS, acute respiratory distress syndrome; BUN, blood urea nitrogen; COP, carbon monoxide poisoning; COHb: carboxyhemoglobin; CAD, coronary artery disease; COPD, chronic obstructive pulmonary disease; DNS, delayed neurological sequelae; ED, emergency department; GCS, Glasgow coma scale; HCT, haematocrit; HFNC, high-flow nasal cannula oxygen therapy; HBOT, hyperbaric oxygen therapy; KDIGO, Kidney Disease Improving Global Outcomes; IQR, interquartile range; IMV, invasive mechanical ventilation; NLR, neutrophil-to-lymphocyte ratio; MAP, mean artery pressure; sCr, serum creatinine; SD, standard deviation; TWBC, total white blood cell.

**Figure 2:**
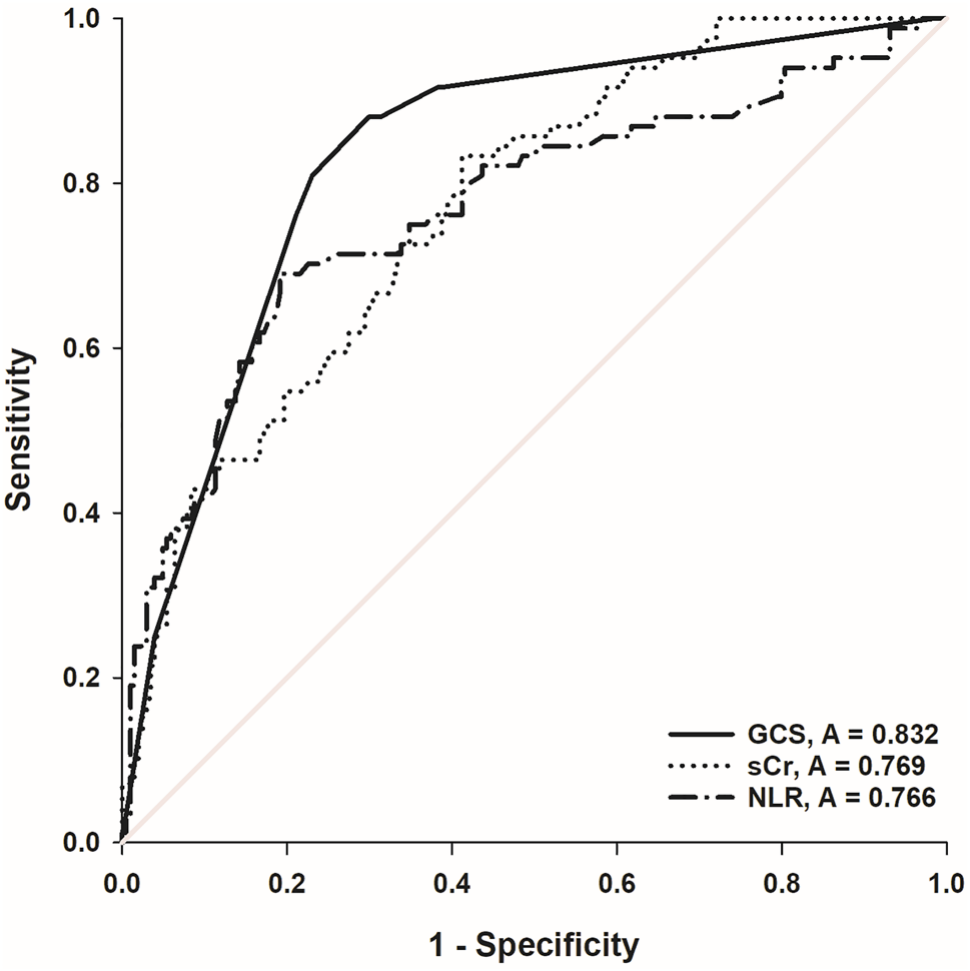
ROC curve analysis of NLR, SCr, and GCS for the frequency of DNS in COP patients. COP, carbon monoxide poisoning; DNS, delayed neurological sequelae; GCS, Glasgow coma scale; NLR, neutrophil-to-lymphocyte ratio; ROC, receiver operating characteristic; SCr, serum creatinine;

The associations of GCS score, SCr, and NLR with DNS in COP patients were further investigated via univariate and multivariable logistic regression analyses. As shown in Table 2, our results showed that the GCS score [odds ratio (OR): 0.548, 95% CI 0.458, 0.655, *P* < 0.001], SCr (OR: 1.088, 95% CI 1.054, 1.123, *P* < 0.001) and NLR (OR: 1.130, 95% CI 1.030, 1.240, *P* = 0.010) were potential predictors independently associated with the DNS after adjustment for confounders. ROC curves of the DNS in COP patients generated using the independent predictors (GCS score, SCr and NLR) are plotted in Fig. 2. As shown in Table 3, the AUCs of the GCS score, sCr and NLR were 0.832 (95% CI 0.784, 0.880, *P* < 0.001), 0.769 (95% CI 0.712, 0.826, *P* < 0.001) and 0.766 (95% CI 0.701, 0.832, *P* < 0.001), respectively. When the optimal cut-off value (maximum Youden index) was 3.745, the sensitivity and specificity of the NLR for the frequency of DNS in COP patients were 0.833 and 0.588, respectively. Meanwhile, the positive likelihood ratio was 2.02, and the negative likelihood ratio was 0.28.

**Table 2:** Independent predictors associated with the frequency of DNS in COP patients by univariate and multivariable logistic regression analysis.

Independent Variable	Univariate		Multivariable	
	OR (95% CI)	*P* Value	OR (95% CI)	*P* Value
Age	1.023 (1.009, 1.038)	0.001		
Male	0.402 (0.239, 0.676)	0.001		
GCS score	0.616 (0.546, 0.695	< 0.001	0.548(0.458,0.655)	< 0.001
TWBC count	1.195 (1.115, 1.281)	< 0.001		
NLR	1.234 (1.151, 1.322)	< 0.001	1.130(1.030,1.240)	0.010
ALT	1.008 (1.000, 1.017)	0.059		
AST	1.007 (1.001, 1.013)	0.024		
SCr	1.064 (1.044, 1.084)	< 0.001	1.088(1.054,1.123)	< 0.001
BUN	1.326 (1.165, 1.510)	< 0.001		
Lactate	1.173 (1.060, 1.298)	0.002		

AST, aspartate aminotransferase; ALT, alanine aminotransferase; BUN, blood urea nitrogen; COP, carbon monoxide poisoning; CI, confidence interval; DNS, delayed neurological sequelae; GCS, Glasgow coma scale; NLR, neutrophil-to-lymphocyte ratio; OR, odds, rate; SCr, serum creatinine; TWBC, total white blood cell.

**Table 3:** Prediction analysis of GCS score, SCr and NLR of the frequency of DNS in COP patients.

Variable	AUC	95% CI	Cut-off value	Sensitivity	Specificity	LR +	LR-	*P* Value
GCS score	0.832	0.784,0.880	8.50	0.701	0.881	5.89	0.34	< 0.001
SCr	0.769	0.712,0.826	67.95	0.691	0.809	3.61	0.38	< 0.001
NLR	0.766	0.701,0.832	3.745	0.833	0.588	2.02	0.28	< 0.001

AUC, area under curve; CI, confidence interval; COP, carbon monoxide poisoning; DNS, delayed neurological sequelae; GCS, Glasgow coma scale; LR + , likelihood ratio positive; LR-, likelihood ratio negative; NLR, neutrophil-to-lymphocyte ratio; SCr, serum creatinine.

To further clarify the predictive value of the NLR in the frequency of DNS in COP patients, 105 consecutive COP patients hospitalised in our institution from January 2021 to June 2022 were recruited for validation, of whom 83 patients met the inclusion criteria (Fig. 1B). The included COP patients in the validation group were divided into two groups (NLR < 3.745 and NLR ≥ 3.745) according to the optimal cut-off value of NLR. As shown in Table 4, the median age of the COP patients was 49.29 ± 19.31 years, and 41.0% (34/83) were male. Overall, the frequency of DNS was 22.9% (19/83), and all patients survived. Significant differences appeared in the frequency of CAD, TWBC count, SCr, BUN, and the frequency of DNS between the two groups. Moreover, the frequency of DNS in the NLR ≥ 3.745 group was notably higher than that in the NLR < 3.745 group [41.4% (12/29) vs. 13.0 (7/54), *P* = 0.003].

**Table 4:** Baseline characteristics and laboratory parameters in the NLR < 3.745 and NLR ≥ 3.745 groups.

Variable	Total (n = 83)	NLR < 3.745 (n = 54)	NLR ≥ 3.745 (n = 29)	*P* Value
Demographics				
Age, mean ± SD, years	49.29 ± 19.31	47.33 ± 17.74	52.93 ± 21.81	0.210
Male, sex, n (%)	34(41.0)	18(33.3)	16(55.2)	0.054
Smoking, n (%)	9(10.8)	6(11.1)	3(10.3)	0.915
Suicide attempt, n (%)	1(1.2)	1(1.9)	0(0)	1.000
Unintentional intoxication, n (%)	82(98.8)	53(98.1)	29(100)	0.461
Time to ED visit, median (IQR), hours	3(1, 7)	2.5(1.0, 6.25)	4 (2, 8.5)	0.201
Chronic comorbidities, n (%)				
Hypertension	6(7.2)	4(7.4)	2(6.9)	1.000
Diabetes mellitus	2(2.4)	1(1.9)	1(3.4)	1.000
CAD	6(7.2)	1(1.9)	5(17.2)	0.033
COPD	5(6.0)	3(5.6)	2(6.9)	1.000
GCS score, mean ± SD	11.63 ± 2.87	11.98 ± 2.62	10.96 ± 3.23	0.125
MAP, mean ± SD, mmHg	97.84 ± 18.56	99.67 ± 20.43	94.41 ± 14.13	0.220
Laboratory parameters				
TWBC count, mean ± SD, × 10^9^/L	8.82 ± 3.40	7.60 ± 2.32	11.08 ± 3.94	< 0.001
ALT, median (IQR), U/L	19.5 (13.8,26.8)	19.8 (13.76,26.73)	17.8 (13.8,29.7)	0.970
AST, median (IQR), U/L	22.1 (17.1,27.1)	22.2 (18.3, 27.26)	21.1 (16.7, 27.3)	0.667
SCr, mean ± SD, umol/L	68.10 ± 24.57	62.43 ± 15.40	78.67 ± 33.79	0.003
BUN, mean ± SD, mmol/L	6.45 ± 2.66	5.85 ± 1.86	7.56 ± 3.50	0.005
Lactate, median (IQR), mmol/L	1.9 (1.1, 2.5)	1.75 (1.1, 2.23)	1.9 (1.2, 3.15)	0.149
COHb, mean ± SD, (%)	33.65 ± 12.06	34.21 ± 9.66	32.81 ± 14.68	0.785
Complications, n (%)				
AKI	16(19.2)	6(11.1)	10(34.5)	0.010
ARF	1(1.2)	0(0)	1(3.4)	0.751
Interventions, n (%)				
HFNC	10(12.0)	9(16.7)	1(3.4)	0.078
HBOT	43(51.8)	29(53.7)	14(48.3)	0.637
Outcomes				
DNS, n (%)	19(22.9)	7(13.0)	12(41.4)	0.003
Hospital stays, median (IQR), days	5.0 (3.0, 7.0)	5.0 (3.0, 7.0)	5 (3.75,8.0)	0.508

AST, aspartate aminotransferase; ALT, alanine aminotransferase; AKI, acute kidney injury; ARF, acute respiratory failure; BUN, blood urea nitrogen; CAD, coronary artery disease; COPD, chronic obstructive pulmonary disease; COHb, carboxyhemoglobin; DNS, delayed neurological sequelae; ED, emergency department; GCS, Glasgow coma scale; HFNC, high-flow nasal cannula oxygen therapy; HBOT, hyperbaric oxygen therapy; IQR, interquartile ranges; MAP, mean arterial pressure; SCr, serum creatinine; SD, standard deviation; TWBC, total white blood cell.

In addition, as shown in Supplementary Table S1, in the validation group, patients showed significantly lower baseline demographic, chronic comorbidity and laboratory data values, including the proportion of smoking, time to ED visit, proportion of hypertension, NLR, and serum lactate, than those in the derivation group. However, the GCS score and SCr in the validation group were notably higher than those in the derivation group. Additionally, the proportion of HBOT in the derivation group was significantly higher than that in the validation group. The length of hospital stay in the derivation group was also notably longer than that in the validation group.

## Discussion

In the present study, the results showed that an increased NLR measured at admission was a significant, independent predictor of the frequency of DNS in COP patients; the NLR exhibited a good predictive value for neurologic outcome in COP patients; and COP patients with an increased NLR showed a significantly higher frequency of DNS in the prospective validation study. Therefore, our findings further extend the evidence of the NLR as a valid and reliable prognostic predictor, proving an increased risk of DNS in COP patients.

The NLR is a widely available biomarker for the immune-inflammatory response and neurological stress. Growing evidence suggests that a higher NLR has been associated with a higher risk of neurologic deficits in patients with neurologic disease, such as acute ischaemic stroke^12^, traumatic brain injury^13^ and spontaneous intracerebral haemorrhage^14^. Recently, the association of peripheral complete blood cell count with neurological sequelae in COP patients was investigated by Moon et al.^15^. They found that the NLR was higher over the first 12 h after admission in patients with a poor neurological outcome than in patients with a good outcome; moreover, the NLR was independently associated with long-term neurological outcome after COP. In contrast to a previous study, we paid more attention to the short-term neurological prognosis of COP patients because these neurological deficits may develop 2–40 days after CO exposure in up to one-third of survivors, and the symptoms are evident by 6 weeks after poisoning^16,17^. The results of our study showed that 103 (27.8%) patients developed DNS by 6 weeks after COP. Moreover, in the derivation group, NLR measured at admission was independently associated with poor neurological outcome of COP patients, with an area under the ROC curve of NLR of 0.766 and a cut-off value of 3.745. Similar findings were demonstrated in the latest study by Gao H et al.^18^. Interestingly, we also found that 86 (29.9%) patients presented with apparent AKI after COP in the derivation cohort, which is consistent with the results of other studies^19,20^. Moreover, COP patients with DNS or increased NLR showed a higher incidence of AKI during the hospital stay. Systemic hypoxia–ischaemia damage, direct damage at the cellular level and inflammation could explain the mechanisms of the development of COP-related nephrotoxicity^20^. However, the association between the development of AKI and prognosis in patients with DNS after COP is unclear, and further study is needed.

In addition, the prognosis of most patients appears to be good, which may be attributed to several factors. First, almost all cases were unintentional poisoning in the present study. A previous study demonstrated that unintentional CO exposure induces most nonfatal poisonings, and intentional CO exposure induces most deaths^21^. Second, public education for high-risk groups provided by the Chinese government is of vital importance.

Classically, CO-associated cerebral injury caused by lipid peroxidation, hypoxic stress, and ischaemic/reperfusion injury has been recognised as an essential feature in the pathogenesis of DNS^1^. Early studies also provided evidence that the inflammatory response is involved in the pathophysiological mechanisms of COP-induced cerebral injury^22,23^. Patients exposed to CO can have activated intravascular neutrophils and can have elevated circulating inflammatory cytokines, leading to cerebral injury and neurological function deficits^23^. Moreover, low lymphocyte counts indicate an increase in the levels of catecholamines and corticosteroids caused by cerebral injury, both of which increase the release of pro‐inflammatory cytokines^15^. In addition, CO-associated cerebral injury may activate the hypothalamic-pituitary adrenal axis, leading to deficiency in the activation of lymphocytes and a decrease in lymphocytes, all of which also suggests the loss of anti-inflammation and neuroprotection induced by lymphocytes^24^. Taken together, these mechanisms might explain why an increased NLR was observed in COP patients with DNS in our study.

The present study has some limitations that must be considered. First, this is a single-centre study with a relatively small sample size (288 in the derivation cohort and 83 in the validation cohort). Second, we collected only one NLR value measured at admission due to missing data. A dynamic change in the NLR during the hospital stay is very important for neurological prognosis as a reliable predictive and prognostic parameter. Third, the primary cause of COP is broadly classified as accidental and intentional intoxication. Almost all cases of COP were unintentional in the present study, limiting the generalizability of our findings. Therefore, future studies of intentional COP are needed for confirmation. Finally, due to the limited clinical data, we did not include clinical parameters in the present study that were missed in half or more of the patients, such as duration of CO exposure, myoglobin, creatine-phosphokinase, and myocardial troponin. Thus, potential selection bias is very likely to exist, and future studies are needed to confirm this hypothesis.

In conclusion, our study demonstrates that a higher NLR measured at admission is associated with the frequency of DNS in COP patients. Therefore, the NLR may be used as a valid and reliable parameter to identify COP patients at risk of neurological deficits and to better guide management strategies.

## Materials and methods

### Patients and selection criteria

The patients recruited in our study were divided into two groups (Fig. 1). One group was retrospectively studied (Fig. 1A). This single-centre, retrospective study was performed at the Emergency Department (ED) of Suqian Hospital of Nanjing Drum Tower Hospital group from January 01, 2015, to December 31, 2020, as a derivation cohort. All adult patients (≥ 18 years) who met the diagnostic criteria of COP (based on clinical history of CO exposure, any clinical signs or symptoms suspected to be related to COP, and elevated carboxyhemoglobin (COHb) level (> 3% for nonsmokers and > 5% for smokers or smoking status was unclear)^19^ and complete clinical data were enrolled. Patients were excluded from this study if they were younger than 18 years of age, had a medical history of haematologic disorder, were transferred from other hospitals, or were lost to follow-up.

Another group was prospectively studied and included consecutive COP patients hospitalised in the ED between January 2021 and June 2022 as a validation cohort (Fig. 1B). The inclusion and exclusion criteria were the same as those of the derivation cohort. Blood samples were obtained from each included patient at admission for haematology and biochemistry detection purposes.

### Data collection

The demographic and clinical characteristics, and laboratory parameters of each patient came from the Electronic Medical Record System at our institution: (1) the baseline demographic and clinical characteristics were: age, gender, smoking, suicide attempt, time to ED visit; chronic comorbidities [hypertension, diabetes mellitus, coronary artery disease (CAD), and chronic obstructive pulmonary disease (COPD)]; Glasgow coma scale (GCS) scores and mean artery pressure (MAP); (2) Laboratory parameters included total white blood cell count (TWBC count, normal reference range: 3.5–9.5 × 10^9^/L); neutrophil to lymphocyte ratio (NLR) were calculated by dividing the neutrophil count by the lymphocyte count; haematocrit (HCT, normal reference range: 35–45%); alanine aminotransferase (ALT, normal reference range: 9-50U/L); aspartate aminotransferase (AST, normal reference range:15-40U/L); serum creatinine (SCr, normal reference range: 57–110 ummol/L); blood urea nitrogen(BUN, normal reference range: 3.1–8 mmmol/L); serum lactate (normal reference range: 0.7–2.1 mmol/L), and carboxyhemoglobin level (COHb, normal reference range: 0.5–1.5%); (3) The complications were acute kidney injury (AKI) and acute respiratory distress syndrome (ARDS). AKI was defined based on both creatinine and output criteria of the kidney disease improving global outcomes (KDIGO) guidelines^25^. Acute respiratory distress syndrome (ARDS) was defined according to the Berlin definition of ARDS^26^; (4) interventions including high-flow nasal cannula oxygen therapy (HFNC), invasive mechanical ventilation (IMV), and hyperbaric oxygen therapy (HBOT) were recorded during hospitalisation; and (5) 28-day mortality and length of hospital stay were also noted. We followed the patients up for prognosis by a telephone interview if the patients were discharged within 28 days. (6) The primary outcome was DNS. DNS was defined as any symptoms or signs of neurologic deficits, such as variable degrees of cognitive dysfunction, mood and movement disorders, and focal neurologic dysfunction, within 6 weeks after COP^1^. Neurologic deficits in the patients were routinely assessed by emergency physicians during hospital hospitalisation, and if the patients were discharged within 6 weeks, a follow-up evaluation for the DNS was performed by a telephone interview. They were also advised to visit the ED again if suspected symptoms or signs occurred during this time.

### Statistical analysis

Statistical analyses of all data in the present study were performed using SPSS 22.0 for Windows (SPSS Inc., Chicago, IL, USA). Continuous variables with normal distributions are presented as the means ± standard deviations (SDs) and were compared using Student's t test. Continuous data with a nonnormal distribution are reported as medians with interquartile ranges (IQRs) and were compared using the Mann‒Whitney U test. Categorical data are reported as absolute numbers and percentages and were analysed using Fisher's exact test or the chi-square test, when appropriate. Independent risk factors associated with the frequency of DNS in COP patients were determined by univariate and forwards stepwise multivariate logistic regression analysis. The variables with *P* < 0.05 from the comparison of demographic and clinical characteristics and laboratory parameters were considered confounders and then entered into the multivariate analysis. The results of the multivariate logistic regression analysis are presented as odds ratios (ORs) and 95% confidence intervals (CIs). Then, we performed the receiver operator curve (ROC) test to evaluate the ability of the NLR to predict DNS in COP patients. A value of *P* < 0.05 was considered statistically significant.

### Ethics approval

This study was conducted in accordance with the Helsinki protocol and standard of Good Clinical Practice, and was approved by the Ethics Committee of the Affiliated Suqian Hospital of Xuzhou Medical University (EC 2021-025).

### Consent to participate

Informed consent was obtained from all subjects and/or their legal guardian(s) after an investigator explained the content of the study.

### Supplementary Information

Supplementary Table S1.

## Data Availability

The datasets used and/or analyzed during the current study are available from the corresponding author on reasonable request.
