# Supplementary material for: The neutrophil-to-lymphocyte ratio is associated with the frequency of delayed neurologic sequelae in patients with carbon monoxide poisoning
Source: Sci Rep. 2023 Nov 11;13:19706. doi: 10.1038/s41598-023-47214-5 (PMC10640581; doi:10.1038/s41598-023-47214-5)
Supplement: Supplementary file 1 — Supplementary Table S1. [file 41598_2023_47214_MOESM1_ESM.docx]

Table S1 Baseline characteristics and laboratory parameters of the derivation and validation groups

| Variable | Derivation group  (n=288) | Validation group  (n=83) | *P* Value |
| --- | --- | --- | --- |
| Demographics |  |  |  |
| Age, mean±SD, years | 53.48±19.29 | 49.29±19.31 | 0.084 |
| Male, sex, n (%) | 116 (40.3) | 34 (41.0) | 0.911 |
| Smoking, n (%) | 79 (27.4) | 9 (10.8) | 0.002 |
| Suicide attempt, n (%) | 4 (1.4) | 1 (1.2) | 1.000 |
| Unintentional intoxication, n (%) | 284 (98.6) | 82 (98.8) | 1.000 |
| Time to ED visit, median (IQR), hours | 8 (5.0,10.0) | 3 (1, 7) | <0.001 |
| Chronic comorbidities, n (%) |  |  |  |
| Hypertension | 56 (19.4) | 6 (7.2) | 0.010 |
| Diabetes mellitus | 12 (4.2) | 2 (2.4) | 0.459 |
| CAD | 9 (3.1) | 6 (7.2) | 0.094 |
| COPD | 12 (4.2) | 5 (6.0) | 0.467 |
| GCS scores, mean±SD | 9.43±3.30 | 11.63±2.87 | <0.001 |
| MAP, mean±SD, mmHg | 96.71±59.20 | 97.84±18.56 | 0.865 |
| Laboratory parameters |  |  |  |
| TWBC count, mean±SD, ×10^9^/L | 9.28±3.97 | 8.82±3.40 | 0.341 |
| NLR, median (IQR), ×10^9^/L | 4.21 (2.38,7.27) | 2.7(1.44, 5.28)) | <0.001 |
| ALT, median (IQR), U/L | 19.45 (14.9,27.0) | 19.5 (13.8,26.8) | 0.886 |
| AST, median (IQR), U/L | 22.9 (17.1,29.85) | 22.1 (17.1,27.1) | 0.650 |
| SCr, mean±SD, umol/L | 62.50±18.08 | 68.10±24.57 | 0.023 |
| BUN, mean±SD, mmol/L | 5.95±2.10 | 6.45±2.66 | 0.074 |
| Serum lactate, median (IQR), mmol/L | 2.0 (1.5,2.88) | 1.9 (1.1, 2.5) | 0.008 |
| COHb, mean±SD, (%) | 29.15±12.53 | 31.38±10.37 | 0.414 |
| Complications, n (%) |  |  |  |
| AKI, n (%) | 86 (29.9) | 16 (19.3) | 0.057 |
| ARDS, n (%) | 8 (2.8) | 1 (1.2) | 0.678 |
| Interventions, n (%) |  |  |  |
| HFNC | 18 (6.3) | 10 (12.0) | 0.078 |
| IMV | 2 (0.7) | 0 (0) | 1.000 |
| HBOT | 225 (88.5) | 43 (51.8) | <0.001 |
| Outcomes |  |  |  |
| DNS, n (%) | 84(29.2) | 19(22.9) | 0.261 |
| 28-day mortality, n (%) | 1 (0.3) | 0 (0) | 1.000 |
| Hospital stays, median (IQR) days | 8.00 (5.00,10.00) | 5.0 (3.0, 7.0) | <0.001 |

AST, aspartate aminotransferase; ALT, alanine aminotransferase; AKI, acute kidney injury; ARDS, acute respiratory distress syndrome; BUN, blood urea nitrogen; COP, carbon monoxide poisoning; COHb: carboxyhemoglobin; CAD, coronary artery disease; COPD, chronic obstructive pulmonary disease; DNS, delayed neurological sequelae; ED, emergency department; GCS, Glasgow coma scale; HFNC, high-flow nasal cannula oxygen therapy; HBOT, hyperbaric oxygen therapy; KDIGO, kidney disease improving global outcomes; IQR, interquartile range; IMV, invasive mechanical ventilation; NLR, neutrophil-to-lymphocyte ratio; MAP, mean artery pressure; sCr, serum creatinine; SD, standard deviation; TWBC, total white blood cell.
